# Short Implant and Heavy Smokers as Predictors for Failure of Immediate Implants: a Retrospective Study

**DOI:** 10.4317/jced.60238

**Published:** 2023-11-01

**Authors:** Alexandre-Marques-Paes da Silva, Ana-Paula-Marques-Paes da Silva, Mayra-Stambovsky Vieira, Antônio Canabarro, Lucio-Souza Gonçalves, Dennis-de Carvalho Ferreira

**Affiliations:** 1 PhD. Estácio de Sá University - UNESA (RJ), Microbiology Department, Rio de Janeiro - RJ, Brazil and Postdoctoral student in the department of Dental Prosthodontics at UERJ - RJ - Brazil; 2 Master in Dentistry at Estácio de Sá University. Faculty of Dentistry, Microbiology Department, Rio de Janeiro - RJ, Brazil; 3 Master in Dentistry. PhD student in Dentistry at Veiga de Almeida University, Rio de Janeiro - RJ, Brasil; 4 PhD. Professor at Veiga de Almeida University, Periodontology Department and School of Dentistry, State University of Rio de Janeiro (UERJ), Rio de Janeiro - RJ, Brasil; 5 PhD. Professor at Estácio de Sá University, School of Dentistry, Microbiology Department, Rio de Janeiro – RJ, Brazil; 6 PhD. Professor at the School of Dentistry, Estácio de Sá University (UNESA-RJ) and Veiga de Almeida University, Microbiology Department, Rio de Janeiro - RJ, Brazil and School of Nursing – UERJ. Rio de Janeiro - RJ, Brazil

## Abstract

**Background:**

The possibility of installing implants in fresh sockets was first proposed as a viable treatment option in the 1970s. Objective: to assess the relationships of subject-level and implant-level characteristics on the failure of immediate implants installed in sites that contained teeth associated or not with chronic apical periodontitis.

**Material and Methods:**

A retrospective study was undertaken with data from patients who received immediate implants with a minimum follow-up of 12 months after loading. The Generalized Estimating Equation, applying a multiple logistic regression model, was employed to investigate the association between predictor variables/co-variables and failure of the immediate implants.

**Results:**

Four hundred and twenty-three implants were installed (208 uninfected/215 infected sites) in 186 patients (92 men/96 women) with a mean age of 57.1 years old. The survival rate of implants was 91%. Approximately half (215/50.8%) of the alveoli that received immediate implants had chronic apical periodontitis associated with the extracted teeth, and 191 (88.8%) of these survived until the last follow-up visit. When the infection-free sites were analyzed, this frequency was higher (93,3%), but the presence of chronic apical periodontitis did not show statistical significance in the implant failure (*p*=0.167). Smokers with a consumption of more than 20 cigarettes/day and short implants had more failures (OR:7.66, *p*=0.012; OR:14.06, *p*=0.002; respectively).

**Conclusions:**

Short implants and consumption of more than 20 cigarettes/day were important predictors for failure of immediate implants, regardless of presence of chronic apical periodontitis.

** Key words:**Osseointegration, dental implant, smokers, study, immediate dental implant loading.

## Introduction

The possibility of installing implants in fresh sockets was first proposed as a viable treatment option in the 1970s (1). Since then, several studies have shown that this is a safe technique that has high survival rates, as well as results that are predicTable and comparable to those with late implants (2). However, questions have arisen regarding the installation of immediate implants in sites containing residual teeth or roots associated with infections in the periapical region (3). Several studies have demonstrated high rates of survival for implants installed in infected sites, including a recent systematic review/meta-analysisthat showed encouraging results ranging from 90.8 to 100% (4).

Teeth indicated for extraction and installation of implants are frequently exposed to infection, which may be of periodontal or endodontic origin (5). Consequently, healing may be affected by a local inflammatory response when installing a dental implant in an alveolus containing pathogenic microorganisms, and, the osseointegration process may fail (6). In such cases, the dentist must follow an adequate approach in order to avoid compromising the osseointegration process and avoid implant failure (7).

Furthermore, certain questions arisen as to whether to keep or not a dental element with periradicular (8), especially since the success rates of primary endodontic treatments, non-surgical retreatment, and periapical surgery are high (9). Currently, there are several possible approaches to teeth with an unfavorable or questionable prognosis due to endodontic involvement (10). However, the decision to maintain or extract a tooth with a doubtful prognosis remains a very controversial issue among dentist (11). Thus, the objective of this study was to assess the relationships of subject-level and implant-level characteristics on the failures of immediate implants installed in sites that contained teeth associated or not with chronic apical periodontitis.

## Material and Methods

-Study design

This retrospective cohort study was previously submitted to and approved by the Research Ethics Committee of the Estácio de Sá University, Rio de Janeiro, under CAAE number 03928818.5.0000.5284 and the data was obtained from medical records and radiological findings of patients at a private clinic in the city of Rio de Janeiro, Brazil, between September 2006 and December 2018. Data collection was performed between February and June 2019. The sample was divided into two groups: comparative group (teeth or roots that did not have chronic apical periodontitis) and study group (teeth or roots that had chronic apical periodontitis).

-Clinical procedure

All the surgeries in this study were done by a single dental-surgeon (specialist in implantology) and carried out under local anesthesia. The teeth were carefully extracted with luxators or forceps after separating roots using rotary instruments, if necessary. A thorough curettage was performed in all sockets in order to remove any granulation tissue (when present), using manual instruments (Curettes) and finally the sockets were irrigated with sterile saline solution (12). According to the implant platform used and in accordance with these recommendations, some implants were anchored 2 to 3 mm intraosseous (13).

Depending on the patient’s individual needs, rehabilitation was carried out with immediate or late loading (after osseointegration) using simple crowns or fixed prostheses. The use of antibiotics prior to the surgical procedure was only performed when necessary (history of mitral valve prolapse to prevent bacterial endocarditis or when there was a request from the doctor accompanying the patient); however, all patients underwent antibiotic therapy after the surgical intervention.

-Inclusion and exclusion criteria

The inclusion criteria were: Single or multiple implants installed immediately after tooth extraction, single-rooted and multi-rooted teeth associated or not with chronic apical periodontitis, with or without periodontal disease and with or without immediate loading; fresh alveolar sites with or without buccal self-contained dehiscence; implants that were subjected to immediate loading with a minimum torque of 45 Ncm; smoking or non-smoking patients; and patients with chronic systemic diseases (hypertension, diabetes, among others), under medical and therapeutic monitoring. Furthermore, there was a minimum follow-up time of 12 months after being subjected to masticatory loading. The exclusion criteria were sites previously healed and with a history of dental implant loss or failure; patients with lack of and improperly filled records regarding the main exposure and outcome variables.

-Sample size calculation

The G*Power 3.1 Program was used to define the sample size, using the following parameters: effect size (w) of 0.30 (Cohen’s Test scale), power of 90% (0.9), α error probability of 5% (α=0.05), for the comparison of two independent groups. The Program estimated a sample size of 158 recipient sites.

-Clinical and radiographic evaluation 

All imaging exams were analyzed by the same operator, who performed the diagnosis, treatment plan, surgical procedure, and prosthetic rehabilitation. The sites were divided into two groups: implants installed in fresh sockets that contained teeth with chronic apical periodontitis (study group) and implants installed in fresh sockets without any endodontic infections (comparative group).

Based on a previous study (14), the presence of chronic apical periodontitis was considered when a radiolucent image associated with the root apex in the image was clearly visible and that these image exams were together with the patients’ medical records (periapical, panoramic, CBCT). In order to confirm the reliability that the image analysis showed an endodontic lesion or not, two different examiners (operator himself and an experienced endodontist) randomly selected 50 images. The Kappa inter-examiner test was performed, and the results obtained were excellent agreement (kappa=0.810).

Patient information was collected from the medical records, as well as data concerning the implants. During follow-up consultations the presence of mucositis or peri-implantitis was analyzed. For this, a clinical and radiographic analysis was performed in order to determine the presence of bleeding in the peri-implant tissue and/or the presence of suppuration through probing (signs of inflammatory reaction) (15). The follow-up radiographs were also analyzed to observe any signs of failure as well as the adaptation of prosthetic work, among other data.

-Outcomes

The primary outcome was to evaluate if the presence of chronic apical periodontitis associated with fresh sockets is a risk factor for the failure of immediate implants installed. Failure was considered when the implant was removed by the operator due fail, at a follow-up consultation or when it was spontaneously lost (15). Implant survival was defined as no clinical signs and symptoms related to the implants that were present (in situ) and functional at the follow-up consultation (15). Also according to another criterion (16), which considered a failure to be early (before the installation of a prosthetic work) or late (after being put into operation). In cases where immediate loading was performed, early failure was considered when it occurred before the first disconnection of the provisional restoration or the first maintenance of the definitive work.

-Statistical analysis

Data analysis was performed using the statistical program Statistical Package for Social Science (SPSS), version 21.0 (IBM, Armonk, NY, USA). The normality of the continuous variables was verified using the Kolmogorov-Smirnov and Shapiro-Wilk tests, in addition to graphical analysis. The qualitative variables were expressed as absolute frequency and relative frequency [n (%)], while quantitative variables were expressed as mean (standard deviation), median (minimum-maximum). Bivariate analyzes regarding the survival and failure of immediate implants were performed using the Chi-square or Fisher’s exact tests for categorical variables, and the Mann-Whitney test for continuous variables, comparing all variables with the outcome studied: survival or failure of immediate implants in infected sites. The Generalized Estimating Equation (GEE), applying a multiple logistic regression model, was employed to investigate the association between predictor variables/co-variables and failure of the immediate implants. The GEE was used to adjust the internal correlation of the observations of each patient’s implant. To analyze the factors associated with the outcome (survival or failure), initially, a bivariate analysis was performed, and variables with *p*<0.20 were selected to perform the multiple logistic regression model (Stepwise Forward method). In the multiple models, variables with multicollinearity characteristics [tolerance <0.1 and the variance inflation factor (VIF) >5 were excluded. The level of statistical significance established was 5% (*p*<0.05).

## Results

This study included 423 immediate implants installed in 186 individuals over the course of 147 months (12 years and 3 months), with an average follow-up time of 39.4 months. The average age of patients described in the records (176 individuals) was 57.1 years old (SD:11.37). Interestingly, 99 (56.3%) of these individuals were below 59 years old, that is, non-elderly, while 77 (43.8%) were 60 or older; however, there was no statistically significant difference between these 2 groups (*p*=0.812) when considering implant failure. Approximately half of the patients were female (50.5%). The majority of the implants (121/70.8%) were installed in individuals who declared that they did not have any systemic changes. The systemic condition did not show significant difference in the bivariate analysis (*p*=0.410) (Table 1).

**Table 1:**
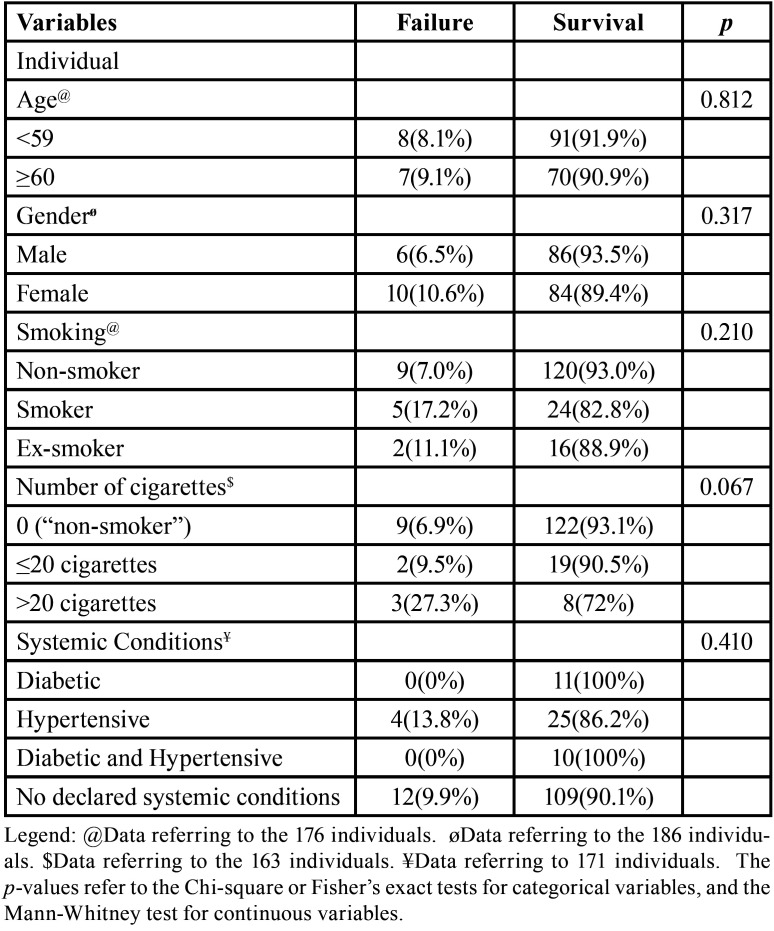
Bivariate analysis between subject-level variables included in the study and the dichotomous failure/survival outcome (186 individuals).

**Figure 1:**
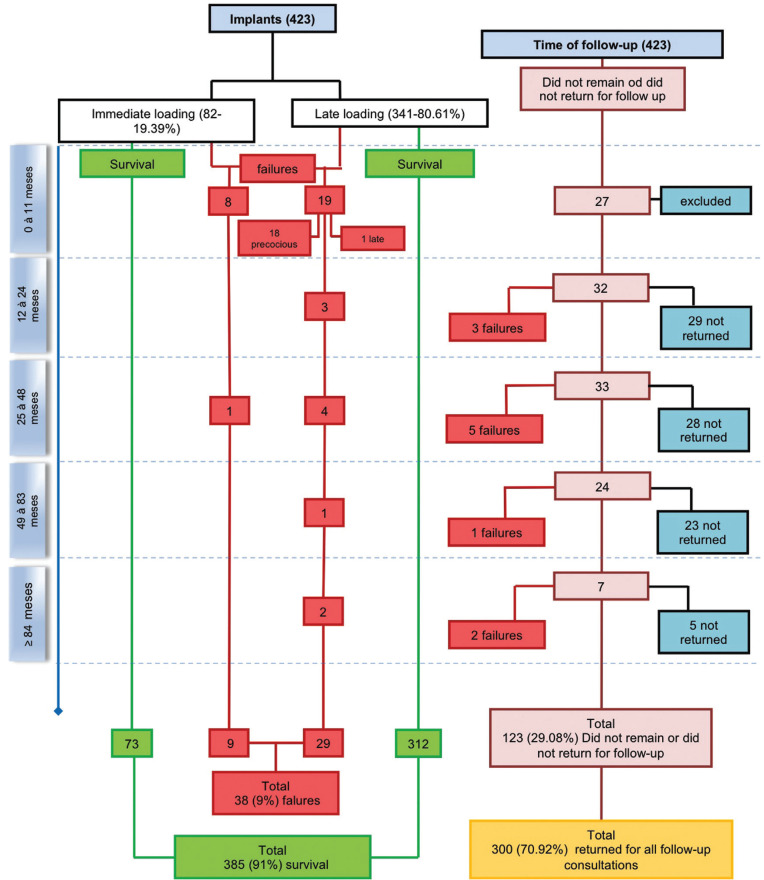
Flowchart of the retrospective study.

The flowchart (Fig. 1) shows that the total survival rate when considering the “implant” as a unit was 91% (385). Only 82 (19.4%) implants received immediate loading, of which 8 (9.8%) failed during the first months of follow-up, even before undergoing the exchange for definitive restoration (0 to 11 months). Although, immediate loading was only performed in cases involving aesthetics, no influence on survival (*p*=0.482) was observed.The Implant survival rate was 93.4% for the first 12 months. Furthermore, by the 84-month follow-up period, 300 (70.9%) implants had returned for follow-up consultations, while 123 (29.1%) had not returned after a certain time or had been excluded from the final sample because they were considered failures. However, when the unit for analysis was the “patient”, total survival was slightly higher, 91.4%. Bivariate analysis was carried out on the prosthetic aspects, such as immediate loading, type of prosthesis, use of a prosthetic intermediary, and adaptation of prosthetic work. Only the latter showed statistical significance (*p*<0.0001) (Table 2); however, it did not show association with failure when applied to GEE analysis.

**Table 2:**
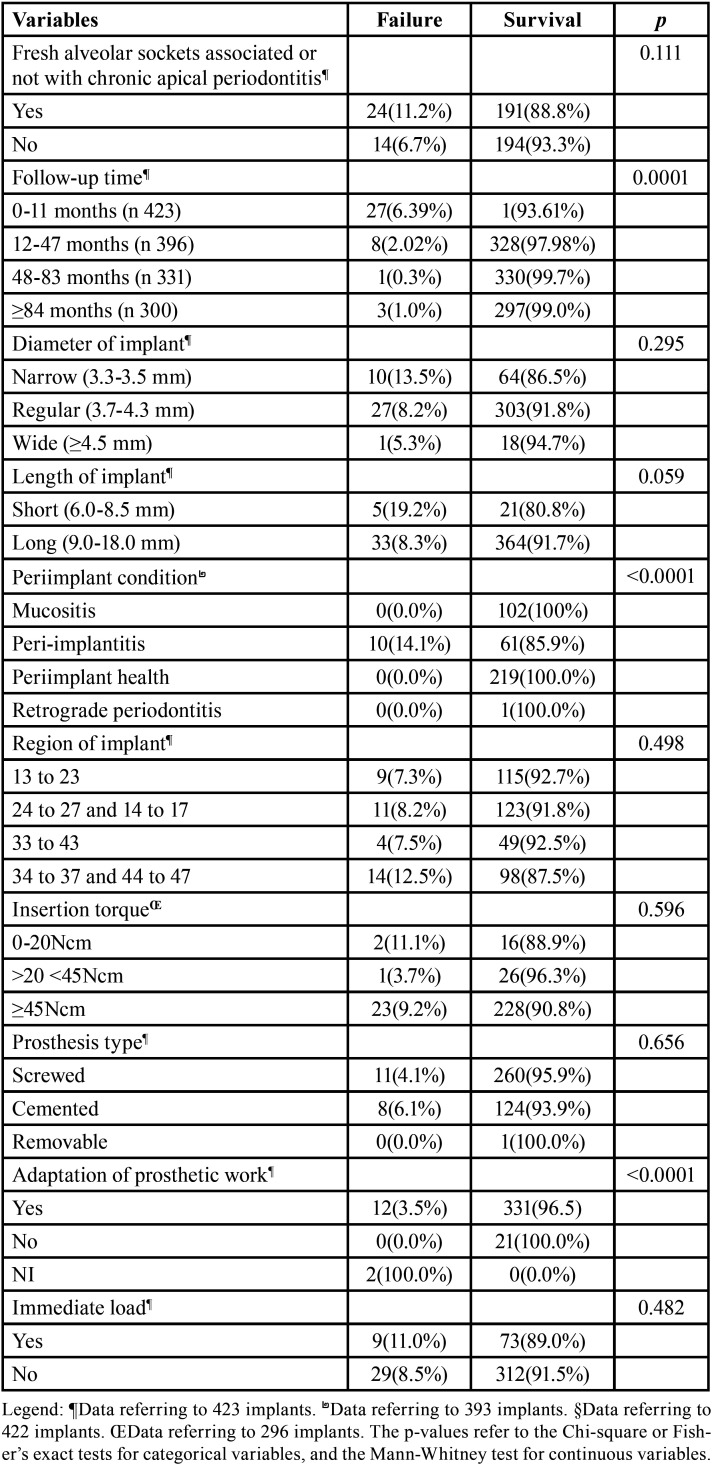
Bivariate analysis between implant-level variables included in the study and the dichotomous failure/survival outcome (423 implants).

More than half of the analyzed implants (57.2%) had peri-implant health until the last follow-up consultation. There were 71 (18.1%) implants, out of the total, with peri-implantitis and 10 (14.1%) of these failed. The average follow-up time for the implants with peri-implantitis that failed was 39.4 months (SD:17.53). All implants that were diagnosed during the follow-ups with mucositis (102/26.6%) survived. There was only 1 (0.3%) case of retrograde periodontitis, where the tooth that was extracted showed endodontic treatment and was associated with an endodontic lesion. In addition, 40 (9.46%) implants did not contain information about peri-implant health. Besides the adaptation to prosthetic work (*p*<0.0001), the peri-implant health (*p*<0.0001) data did not enter the GEE analysis, as they presented multicollinearity characteristics (Table 2).

Three variables were included in the GEE, applying a multiple logistic regression model: number of cigarettes consumed (*p*=0,012), length of the implants (*p*=0.002) and the presence of endodontic lesion (*p*=0.167).

Approximately half (215/50.8%) of the alveoli that received immediate implants had chronic apical periodontitis associated with the extracted teeth and 191 (88.8%) of these survived until the last follow-up visit. When the infection-free sites were analyzed, this frequency was higher (93,3%), but the presence of chronic apical periodontitis did not present any statistical significance in the survival (*p*=0.167). On the other hand, the length of the implant had a direct relationship with failure. The “short” implants were 14.06 times more likely to failure compared to the “long” implants (*p*=0.002). The majority of patients declared that they did not smoke, while the rest of the sample studied was divided into two groups: consumption of 0 to 20 cigarettes (12.9%) and those that smoked more than 20 cigarettes/day (6.7%). When “smokers who consumed more than 20 cigarettes/day” were compared with “non-smokers”, the risk of implant failure was seen to be 7.66 times greater (*p*=0.012) (Table 3).

**Table 3:**
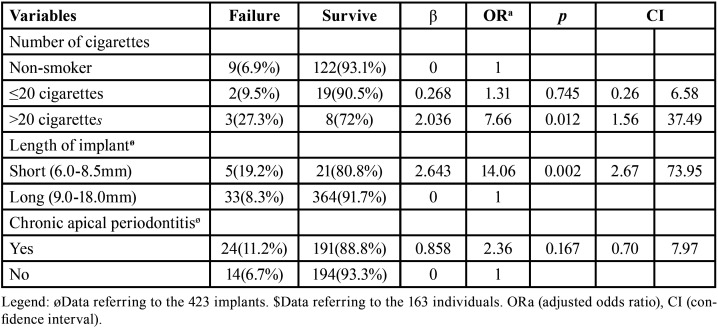
Multiple logistic regression analysis to investigate the association between predictor variables/co-variables and failure of immediate implants.

## Discussion

This study analyzed 423 immediate implants installed in 186 individuals with a mean age of 57.1 years old. The cumulative survival rates over 147 months were 91% and 91.4% (implants and patient units, respectively). These rates are in line with those found in previously published studies, where rates higher than 90% were observed, demonstrating the safety of these immediate implant installation techniques (2,17). A possible explanation for the fact that the survival rate was not higher may be the fact that all surgeries were performed by the same dental surgeon, that is, the same professional over the years mastered surgical techniques better and acquired more experience in implantology. However, when only sites with the presence of chronic apical periodontitis were analyzed, this survival rate decreased [191(88.8%)/215]. Even so, no significant difference both in the bivariate and GEE analysis was observed. There are reports in the literature that periapical pathosis may not be contra indicated if the socket is debrided and disinfected effectively (18). However, other authors advise against the installation of immediate implants under these conditions, and a meta-analysis demonstrated that the immediate instalation of implants in infected cavities tends to increase the risk of implant failure (4,18).

Smoking can be considered a negative factor in wound healing and may be a risk factor for the survival of implants (19). In this study the implants installed in individuals who consumed more than 20 cigarettes/day had 7.66 times more risk of failure when compared with individuals who did not smoke (OR:7.66, CI: 1.56-37.49, *p*=0.012). As in this study, another study evaluated smoking as a risk factor, subdividing the group of smokers (non-smokers; light smokers and heavy smokers). However, unlike the present study, smoking was considered a risk factor for implant survival, and the relative risk of heavy smokers compared to non-smokers was also high, 21.8 (19). Other studies have found no significant correlation between smoking and implant survival (16).

The bivariate analysis showed that the follow-up time presented statistical significance and 300 (70.9%) implants remained in the reassessment consultations for up to at least 84 months. The follow-up appointments are very important to evaluate the results of dental therapies, including dental implant treatments, that they should not be limited to their installation, but should also include a clinical and radiographic monitoring protocol with the patient at predetermined time intervals. Such follow-up procedures can help prevent biological complications and attain higher long-term survival rates (20).

The length of the implant entered the GEE analysis, and it had a direct influence on the survival of the implants. Short implants were 14 times more risk to failure when compared to long implants (OR:14.06, CI: 2.67-73.95, *p*=0.002). Some studies, contrary to our findings, have not shown significant differences in survival rates between short and long implants (21). Thus, it is important to carry out a thorough assessment of the length and shape of the roots to be extracted, since the implants must be anchored in the most apical region of the socket, anchored 2 to 3 mm into the apical bone where the implant is to be anchored (13,22). Bearing in mind that the implants analyzed in this work were all immediate, which may justify the fact that the number of long implants (397/93.9%) was much higher compared to the number of short implants (26/6.1%).

The bivariate analysis of the results showed that the peri-implant condition had a negative impact on the survival of the implants. However, when mucositis was observed (102/26.2%) at follow-up visits, 100% of them survived over the study period, while 10(14.1%) out of 71 cases of peri-implantitis, failed. These findings corroborate with another study with similar survival rates where mucositis was detected in 33% of the analyzed implants and peri-implantitis in 16% (23). Peri-implantitis is considered, by many authors, as one of the main causes of late failures implants due to bone loss, which can begin at these locations. So, regular monitoring and support programs for patients who receive implants results in greater survival of implants over time (24).

Various authors have reported that implant failures in maxilla are more common compared to the lower region, and this can be justified by the fact that the maxillary bone density is lower compared to the mandible region, especially in the region of the upper posterior teeth (25). In this study there was no statistically significant difference between maxilla and mandible. A possible reason for this is that all the implants were performed by the same surgeon, who used the same technique. Furthermore, most of the implants (296/84.8%) analyzed that contained the torque value information in the respective patients’ files reached an insertion torque ≥45Ncm, guaranteeing good primary stability (13). This fact may justify the lack of any statistical significance when evaluating the implant insertion torque. However, there are studies in the literature in which the arch is not a risk factor for the survival of the implants (26). Researchers analyzed 721 implants installed in 296 patients and found that the losses in the upper arch presented statistically significant higher values (25). In contrast to this result, other authors found no significant differences when evaluating 107 implants installed in 106 patients, with 53 in the upper arch and 54 in the lower arch, and with only one loss in the mandible region (27).

Among the limitations of this study is that some of the medical records did not contain all the information properly filled in and others depended on the patient’s declaration (24). Other limitations also included: the number of missing teeth, use of bone graft material and membranes, bone volume at recipient sites that were not assessed, the type of healing was not specified (submerged or transmucosal) and, finally, the bacterial platelet index was not described in the patients’ records. Also, some factors that may increase the risk of failure, such as habits other than smoking, previous periodontal conditions of the extracted and remaining teeth, average size of the apical lesion, as well as the oral hygiene conditions of the patients that were not taken into consideration (24).

## Conclusions

The findings of our study suggest that short implants and the consumption of more than 20 cigarettes/day were important predictors for failure of immediate implants. These reinforce the fact that the installation of implants in infected sites is a promising option with predictable results. However, new prospective clinical studies with pre-determined protocols and maintenance programs with follow-up consultations must be performed.
